# Experienced Nurses' Motivation, Intention to Leave, and Reasons for Turnover: A Qualitative Survey Study

**DOI:** 10.1155/2023/2780839

**Published:** 2023-09-16

**Authors:** Anna Hörberg, Christian Gadolin, Maria Skyvell Nilsson, Petter Gustavsson, Ann Rudman

**Affiliations:** ^1^School of Health and Welfare, Dalarna University, Falun, Sweden; ^2^School of Business, Economics and IT, University West, Trollhättan, Sweden; ^3^Department of Health Sciences, University West, Trollhättan, Sweden; ^4^Department of Clinical Neuroscience, Karolinska Institute, Stockholm, Sweden

## Abstract

There is a global nurse shortage, and researchers have made great efforts in trying to unveil the reasons for turnover and how to increase retention. However, such research has had a tendency to study variables related to intention to leave (ITL) or turnover as isolated phenomena. *Objective*. To simultaneously explore what factors motivate experienced nurses in the workplace and the underlying reasons for strong ITL and high staff turnover within the profession. *Design*. An inductive qualitative content analysis was used based on data from open-ended survey questions. The data originated from the longitudinal analyses of nursing education/employment/entry (LANE) in work-life study. The qualitative data analyzed in this study were distributed in October 2017–January 2018, to all nurses in three cohorts corresponding to 11-, 13- and 15-year postgraduation. Of the 2,474 nurses answering the survey, 1,146 (46%) responded to one or more of the open-ended questions. *Results*. The result showed that what motivates experienced nurses, their intention to leave (ITL), and reasons for turnover could be described in the form of five broad categories, namely, organizational characteristics, work characteristics, relationships at work, work recognition, and health issues. There was rarely a one single reason described, rather several reasons needed to be experienced over time for nurses to stay motivated or leave the profession. *Conclusions*. There is no single reason that makes nurses leave the profession, nor is there one single reason that makes them motivated to stay. Retention and turnover are complex processes and need to be addressed as this, not as a single isolated phenomenon.

## 1. Introduction

Nursing is defined by autonomous as well as collaborative care with the aim of preventing illness and nursing sick, injured, disabled, or dying people of all ages. Moreover, nursing encompasses caring and advocating for families and loved ones, shaping healthcare policy, management, education, and research [1, 2]. Since the start of the COVID-19 pandemic in 2020, the vital role nurses play in society and healthcare systems worldwide has become more evident than ever before [3]. Today, nurses make up over half of all healthcare professionals and are often the first, and sometimes the only healthcare professional a patient will meet, which further highlights the significance of nurses in the healthcare sector [4–6].

Nurses perform a plethora of tasks and care for people in a multitude of healthcare settings, often shouldering a high workload and working long hours [7]. During the last decade, increased staff turnover among nurses has led to a global needs-based shortage of nurses [6, 8]. Global efforts have been made to increase the number of nurses, including various local retention strategies [9, 10] and increasing the number of places in nursing programmes. Despite these efforts, the World Health Organization (WHO) forecasts a shortfall of almost 6 million nurses by the year of 2030 and emphasises the importance of retaining experienced nurses in the workforce [6]. For some time now, researchers have made great efforts to try to understand the reasons why nurses leave the profession.

Studies looking at why nurses leave the profession often focus on intention to leave (ITL) or the underlying reasons for staff turnover [8, 11]. ITL is clearly a complex issue triggered by organisational factors, such as the work environment, leadership, staffing levels, and the state of working relationships [12, 13], as well as individual factors such as stress, burnout, and a sense that the work nurses perform is not valued [13, 14]. Earlier studies have revealed that turnover is driven by management style, highlighting a lack of good visible leaders, high workload, stress, disempowerment, and/or lack of autonomy [8, 15]. Previous research has shown that the final decision of nurses to leave the profession (turnover) is often proceeded by a lengthy period of consideration, or ITL [16]. It therefore seems unviable to study these variables independently.

One way to understand retention and/or turnover is Herzberg's two-factor theory, which relates job satisfaction/dissatisfaction to motivation [17]. According to the theory, there are two dimensions to job satisfaction: hygiene and motivation. Hygiene factors are essential to maintain a pleasant workplace, and their absence will lead to dissatisfaction; meanwhile, motivation factors can create satisfaction by fulfilling the employee's needs for personal growth and a sense of meaning [18]. In the context of nursing, hygiene factors include a manageable workload, sufficient staffing levels, and reasonable salary and benefits, while motivators include a diversity of tasks, stimulating responsibilities, and seeing patients recover [19, 20]. Although the two-factor theory has proved useful in exploring job satisfaction and retention in nursing, it has also been found that, when describing their motivators, nurses contradict the basic tenets of the theory [19]. This suggests that further research on motivation may be beneficial in terms of understanding retention.

Given the global shortage of nurses and the general high level of work-life stress in the nursing profession, there is every reason to increase our understanding of all aspects that influence nurses to remain in or leave the profession. Rather than seeking to understand motivation, ITL, or turnover as isolated phenomena, this study aims to simultaneously explore what factors motivate experienced nurses in the workplace and the underlying reasons for strong ITL and high staff turnover within the profession. This approach may help further understand both the turnover and retention of nurses.

## 2. Materials and Methods

The data used here originate from the longitudinal analysis of nursing education (LANE) study [21]. The LANE study is a prospective national study in which three Swedish national cohorts (2002, 2004, and 2006) of nurses were repeatedly surveyed early in their career, with a follow-up 11–15 years after graduation. The qualitative data analyzed in this study were collected through open-ended questions in a survey distributed between October 2017 and January 2018 to all nurses in the three cohorts, i.e., 11, 13, and 15 years after graduation.

### 2.1. Sample

Of the 2,474 nurses answering the survey, 1,146 (46%) responded to one or more open-ended questions and thus constituted the sample for this study.

### 2.2. Data Collection

Initially, all nursing students enrolled at the 26 Swedish universities offering nursing programmes graduating in the autumn of 2002, 2004, and 2006 were invited to participate in the LANE study. A total of 4,316 graduates consented to participate when invited, and 4,002 were still eligible for participation when the long-term follow-up (11–15 years after graduation) was conducted in 2017. The response rate at the long-term follow-up was 62% (2,474). Data used in this study were collected from three open-ended questions included in the long-term follow-up questionnaire. The three questions addressed (a) engagement and motivation, (b) ITL, and (c) reasons for leaving the profession. The open-ended questions and the number of answers in the form of written comments to each question are presented in Table 1. In total, 1,146 participants gave 1,542 answers to the questions (29 participants answered all three questions, 338 answered two questions, and 779 answered one question).

### 2.3. Data Analysis

The analysis process followed the inductive qualitative content analysis framework presented by Graneheim and Lundman [22]. The written comments to the three questions (Table 1) constituted the unit of analysis and were initially treated as three subject areas: professional work motivation, why nurses intend to leave, and why nurses have left the profession/turnover. All the written comments were scanned and digitally transferred to the data analysis program NVivo [23]. The primary responsibility for analysing different subject areas was shared among the researchers. CG and MSN analysed the text about motivation, while AH and AR analysed the text related to ITL and turnover, all following the analytical steps outlined below. In the first step, the written comments were read and meaning units—the relevant text for the purpose of the study—were identified. In the second step, the meaning units were coded. Coding involved labelling the meaning units with a descriptive word close to the original text [24]. In the third step, the codes were abstracted and sorted into subcategories. These three steps were followed by a comparison of the two research groups' preliminary results. The purpose of this comparison was to identify similarities and differences related to the three subject areas. During this process, the labelling and definition of five categories could be completed (Table 2). Finally, each category could be described based on what emerged in all three subject areas.

### 2.4. Ethical Considerations

Approval for the study was received from the Regional Research Ethics Committee at Karolinska Institutet, Stockholm, Sweden (ref. no. 01–045), and the Regional Ethics Review Board in Stockholm, Sweden (ref. no. 04–587 and ref. no. 2016/793–32). All respondents in the study gave informed consent to participation. A cover letter emphasising the voluntary nature of participation and the option to terminate participation in the study at any time was attached to the survey, as was information on how to contact the research team. All participants gave informed consent.

## 3. Results and Discussion

Among the respondents, 1,035 (90%) were female, and the age distribution was as follows: 504 (44%) were younger than 39 years, 391 (34%) were aged 40–49 years, and 251 (22%) were older than 50 years. It had been between 11 and 15 years since they graduated from nursing education. In addition, 57% of the respondents had received specialist nurse education, and they worked across various sectors of the healthcare system, including inpatient care, outpatient care, home care, prehospital ambulance care, research, and management.

**Figure 1:**
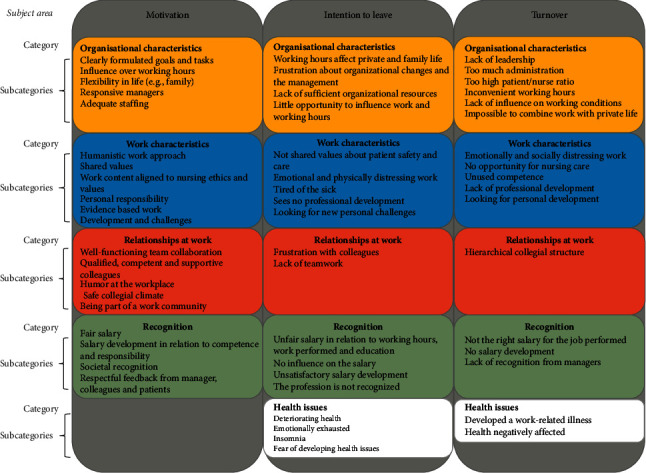
Illustration of the result including subject areas (horizontal), categories, and subcategories (vertical).

The result showed that what motivates experienced nurses, what sparks their ITL, and reasons for high turnover could be divided into five broad categories: organisational characteristics, work characteristics, relationships at work, work recognition, and health issues (Figure 1). The three subject areas motivation, ITL, and turnover are illustrated horizontally, and descriptions of the categories and subcategories within each of the three subject areas are illustrated vertically.

It was rare for a single motivator or underlying cause for ITL or turnover to be stated. Motivation often seemed to be linked to several accumulating factors. Similarly, a combination of reasons was often stated by nurses who either intended to leave or had already left the profession. Although the content of the categories interacts in the interests of clarity, the categories will be described separately. Verbatim quotes related to each subcategory can be found in Table 2.

### 3.1. Organisational characteristics

#### 3.1.1. Motivation

Nurses were motivated when the organisation clearly defined roles, goals, and tasks. Another motivator was when management offered employees opportunities to influence routines and work schedules. This was particularly true of scheduling working hours, which enabled nurses to better plan their personal lives and achieve work-life balance, both between shifts and when not scheduled to work. A manager's ability to translate overarching organisational objectives into tangible and specific daily goals was also an important motivator. Those who could do this and reorganise work when workloads became excessive and ensure adequate staffing were described as responsible managers.

#### 3.1.2. ITL

Organisational characteristics could also be found in data regarding why nurses often thought about leaving the profession. These included working hours, the difficulty of combining work with family or personal life, lack of time for recovery, and frustration with management and the organisation of work. The nurses also described a lack of organisational resources, including inadequate staffing levels. The results of the analysis also revealed that nurses who felt unable to influence their work often considered leaving the profession.

#### 3.1.3. Turnover

Among the organisational characteristics referred to by the nurses who had left the profession were poor leadership, lack of influence, and inadequate staffing levels. These nurses described a lack of leadership and a lack of trust in their immediate manager, as well as discontent with constant reorganisation. Excessive administrative demands and a high nurse-to-patient ratio were described as draining. The decision to leave was also made due to demanding working hours that precluded a healthy work-life balance. More specifically, a schedule with too many short turnarounds with inadequate rest, too many shifts in a month, or excessive overtime were stated as the reasons for high staff turnover, as was a lack of foresight regarding holidays and scheduling that made it impossible to combine work with family life.

### 3.2. Work Characteristics

#### 3.2.1. Motivation

Work characteristics described as motivational included being able to nurse based on a humanistic approach and in line with the nurse's own personal values. This was described as a matter of considering and protecting the patient's dignity and integrity throughout the care situation. Another motivational factor was being afforded a large measure of professional autonomy. Autonomy was described as the opposite of when tasks were controlled in detail by others. Nursing was also considered motivational when the nurse was able to take responsibility, both individually and as a member of a team, for achieving operational goals, developing nursing, and providing high-quality, evidence-based care. Continuing professional and personal development was also a motivator.

#### 3.2.2. ITL

Nurses with intention to leave the profession also highlighted specific work characteristics. Several nurses described their duties as not being in line with nursing ethics and values. The issue of patient safety was raised, such as when managers and nurses had different thresholds for when patient care could be considered safe. They highlighted moral distress experienced when unable to meet the personal needs of patients and their loved ones by providing high-quality care. Work factors were described as emotionally and physically demanding; caring for the sick had begun to drain them. They also noted that the nature of their work left no opportunity for desired personal and professional development. Nurses described changing job or profession as one way of acquiring this sought-after development.

#### 3.2.3. Turnover

Nurses who had left the profession described the job as unsustainable by nature due to the weight of emotional and ethical distress. They also stated that there was too little focus on patient safety and providing high-quality nursing care. One major reason for turnover was the perception that caring had “disappeared” from nursing, their competence as nurses was not being utilised, and they were therefore unable to provide patients with high-quality care. Nurses who had left the profession also stated that there was little opportunity for professional development and that they wanted to develop both personally and professionally.

### 3.3. Relationships at Work

#### 3.3.1. Motivation

Good relationships and well-functioning teamwork among competent, qualified, and supportive colleagues were stated as important motivational factors. A supportive, collegial climate was also described as facilitating continuous learning and development and thus as particularly motivational. The important qualities in a relationship included being able to express one's thoughts, feelings, and opinions freely and to share laughter with colleagues even when work was stressful or emotionally taxing. A safe, tolerant, and inclusive collegial climate that provided a sense of belonging was also a motivation. It was also considered important that equality should be an accepted part of the work community.

#### 3.3.2. ITL

Frustration with collegial relationships was mentioned as one reason why nurses often thought about leaving the nursing profession. That said, these comments consisted of short statements that lacked further detailed information. Although brief, many comments concerned a lack of teamwork and a lack of trust and collaboration with the colleagues closest to the respondents.

#### 3.3.3. Turnover

Nurses who had left the profession mentioned the professional hierarchy as a reason. One cause of hierarchical tension was when the nurse believed that they were considered to be doing no more than following orders given by doctors.

### 3.4. Recognition

#### 3.4.1. Motivation

Recognition is clearly important for sustaining motivation at work, including in terms of being paid a reasonable living wage. The factors that nurses included in the definition of a reasonable wage included individual salary setting, as well as the profession's salary level. It was considered motivating when the salary was set fairly taking into account individual performance and hard work, unsociable working hours, competence, and training. Remuneration for additional responsibilities or developing work on the ward was perceived as having a motivational effect. Moreover, a salary commensurate with the important societal function of the nursing profession was seen as important to sustaining motivation. It was also considered motivational when, in addition to any personal recognition nurses might receive, the significance of nursing as a profession was acknowledged at governmental and societal levels. For example, nurses described themselves as motivated when political decisions concerning the organisation of healthcare including the nursing perspective, or when the mass media covered the work of nurses in an insightful way. Recognition at a personal level was described in terms of nurses being treated with respect, receiving positive feedback, and feeling appreciated by patients, colleagues, or management.

#### 3.4.2. ITL

One frequently stated reason why nurses often thought about leaving the profession was a lack of recognition, particularly in terms of salary. Salary was described as unfair in relation to the workload, commitment, unsociable working hours, and short shift turnaround. Furthermore, when the salary was not perceived as compensating for the price nurses pay in terms of their family and personal life, they began to consider leaving the profession. There were descriptions of salaries not rising despite efforts such as further education, taking on extra assignments, or showing commitment. Aside from salary, lack of recognition from colleagues, patients, managers, and society was also mentioned as the reason to consider leaving the profession.

#### 3.4.3. Turnover

In terms of recognition, the nurses who had left the profession did name the lack of a fair salary, salary development, or recognition from managers as a reason for leaving the profession.

### 3.5. Health Issues

The fifth category, health issues, was represented in two of the subject areas (ITL and turnover).

#### 3.5.1. ITL

Common reasons behind ITL were deteriorating health, emotional exhaustion, and/or insomnia. Some nurses who often thought about leaving stated that, although their health had not yet been affected, they were afraid that their health would suffer if they continued working in nursing.

#### 3.5.2. Turnover

Stress, insomnia, and fatigue were described as contributing factors to deteriorating health and a reason for leaving. From some of the comments, it was clear that, while the nurses themselves did not want to leave the profession, they felt that this was the only way to take control of their health and sustain a personal life.

## 4. Discussion

To the best of our knowledge, this is the first study to simultaneously explore motivation, ITL, and turnover in the context of nursing. The current view of staff turnover in nursing is that it is the result of a negative spiral that ultimately ends with nurses abandoning the profession [25], hence Selberg and Mulinari's [25] concept of turnover contagion; one nurse's decision to leave a failing clinic prompts other nurses to follow suit. In other words, “quitting leads to quitting” (p.96). By addressing all four areas in which, according to the result of this study, there seems to be an interrelation between motivation, ITL, and turnover, we may be able to not only halt a negative spiral but also increase motivation and, in the long term, wellbeing in the workplace.

Taken together, the five categories—organisational characteristics, work characteristics, relationships at work, recognition, and health issues—are crucial for sustaining motivation and avoiding staff turnover. This study reveals that while organisational characteristics can be highly motivating, they can also be the underlying causes of ITL and staff turnover. Judging by the result of this study, organisational characteristics can motivate nurses even when involving a high level of responsibility and challenge; however, this is highly dependent on management and leadership. This is in line with the previous research showing that nursing leadership plays a vital role in creating healthy work environments and manageable workloads [12, 26, 27]. Nurses in leadership positions also play an important role in ensuring that their fellow nurses have control over their work and shifts and have sufficient time for recovery [28, 29], as well as authorising work schedules that take account of the individual nurse's life situation [26, 30]. The organisational characteristics category clearly showed the underlying problems in terms of affording nurses the possibility to influence their own work and working hours and to recover between shifts. Furthermore, the result of this study clearly shows that nurses that are unable to achieve a healthy work-life balance find it difficult to sustain motivation and remain in the profession.

In the category work characteristics, nurses stated that meeting and caring for patients were highly motivational, thus revealing a true passion for the profession of nursing. Even among those who intended to leave or had left, there was clearly a strong wish to continue nursing; however, according to their accounts, the situation made it impossible to provide care in a manner consistent with their professional values. Also, in the category of work characteristics, nurses who had left the profession gave emotional distress as one of the reasons for doing so. Nursing is clearly demanding work, and the negative impact of emotional stress on the wellbeing of nurses has been thrown into stark relief by the COVID-19 pandemic [3, 5]. It could be argued that the stress that nurses experience is inherent to the profession, given the highly unpredictable nature of the job and the unsociable working hours nurses must work in order to provide healthcare around the clock. To make such demanding work characteristics motivational, and so that nurses can thrive in their jobs, the emphasis should be on providing adequate resources and individual control over their work [31]. Toode et al. [30] underline that even a more intense workload may be motivating when it leads to an increased sense of professional accomplishment, career opportunities, or professional development. This reasoning is in line with the findings of another Swedish study conducted by Gadolin et al. [32], which showed that when nurses perceived that their contribution was valued and their wellbeing was being safeguarded, they could focus on their main task of providing care.

Furthermore, Gadolin's results [32] reveal the importance of validating nurses' sense of competency and utilising their competence in the role of nurse. The results of our study also show that when nurses perceived their nursing competence to be valued and appropriately utilised by the organisation, it positively influenced their working experience. One core characteristic of nursing has been said to be that of “caring about” patients and their loved ones by providing psychosocial support, compassion, and sympathy [33]. At the same time, paradoxically and partly contrary to these earlier studies, our study shows that nurses did not feel that their competence was being fully utilised; in other words, there is a discrepancy between what nurses have the ability to do and what they were permitted to do. It has been argued that the lingering and obsolete traditionalist view of nurses as being merely the physician's assistant continues to play down and negatively affect the expertise of nursing practice [34]. In the relationships at work category of this study, these views were described in terms of hierarchical collegial relationships and as one reason for turnover. More and more noncaring tasks, such as cleaning and administration, have also been described as stealing valuable time away from caring [33]. Incongruity between the nurse's professional values and competence and tasks they are expected to perform may leave them caught in-between and faced with ethical dilemmas [35].

The category relationships at work also highlight how important relationships in the workplace and being able to rely on the competence of colleagues are to motivation and remaining in the profession. Management in workplaces perceived as healthy organisations is described as creating a culture of openness, respect, fairness, and justice [36]. Coomber and Louise Barriball [37] describe poor relationships with colleagues as one of the most significant factors behind work-related stress, leading to ITL and turnover. The social aspects of the workplace, such as being able to share and discuss opinions and concerns about patient care with colleagues, have been found to be associated with empowerment, thus reducing the likelihood of nurses leaving the profession [12]. Furthermore, good collaboration, social support, and positive team spirit on a ward where nurses are treated as equally valued healthcare professionals has been highlighted as important motivators [30]. Although interpersonal relationships are presented as hygiene factors in Hertzberg's two-factor theory [18], the result of our study, in line with prior research [19, 30], concludes that nurses describe good collegial relationships as motivational factors that contribute to job satisfaction and motivation for retention. Gadolin et al. [32] recently demonstrated that sound relationships with colleagues are vital if nurses are to feel supported by the organisation, which in turn is associated with a variety of favourable outcomes such as greater commitment and improved wellbeing [38]. It is also a prerequisite if quality improvement work in healthcare is to succeed [39]. So, the results of this study support previous studies in underlining the importance of collegiality in healthcare.

The results of the present study further highlight the importance of recognition to the work of nurses, and this was emphasised in all subject areas. One of the key aspects of recognition is a fair and adequate salary, and the study shows that nurses' salaries are frequently perceived as both unfair and inadequate. According to the findings in the review by Coomber and Louise Barriball [37], one often-stated concern regarding nurses' salaries was the perceived inequity of pay given the high level of knowledge, responsibility, and workload compared to “less qualified professions.” Experienced nurses also felt that their level of experience was not recognised as important [37]. Recognition of such factors, or lack thereof, in a nurse's pay packet can either be a motivator or lead to ITL and staff turnover. Again, the result of this study contrasts Hertzberg's two-factor theory [18] as salary and monetary compensation are hygiene factors. The result of the present study indicates that recognition in terms of a fair salary can be motivation factors as it signals that nurses are recognized as important. That said, it is important to emphasise that, in line with prior research, this study demonstrates that while salary was often stated as one reason for ITL or leaving the profession, it was rarely the sole source of discontent; nurses also demanded other forms of recognition [12].

Although health issues were only raised in two subject areas, they were described as a major obstacle to remain in the profession, prompting ITL and departures. This confirms the results of an earlier quantitative study showing that early-career burnout left nurses feeling disengaged and exhausted at work, leading to higher [14] and escalating levels of intention to leave the profession [40]. Moreover, there were increasing symptoms of burnout, while undergraduates predicted nurses' ITL and turnover after graduation [41]. Interestingly, this qualitative study based on nurses' own words raises similar issues regarding fear of or actual deteriorating health. It is clear that, while health was not considered a motivational factor at work, the deleterious effects of the job on health were considered too high a price to pay for remaining in the profession. Hence, employers taking preventive measures that detect the early signs of burnout in order to reduce staff turnover is important.

### 4.1. Limitations

Among respondents who stated that they had left the nursing profession, some had moved on to other roles in healthcare such as university teachers, ward managers, or working with healthcare development. As these are vocations in which Sweden clearly needs nursing competence, one must question why these nursing graduates felt that they had left the profession. Further research is needed to investigate whether nurses themselves see a distinction between leaving the profession and carving out a career elsewhere in healthcare, such as in academia or management: is remaining “in the profession” solely associated with clinical practice?

In this study, data quality did not allow for coding of the “underlying” meaning (i.e., it was not considered possible to derive any themes). Therefore, the analysis resulted in a descriptive manifest level of the data [24]. The quality of data varied from concise answers to more expansive descriptions. At times, it was problematic to separate mutually exclusive categories, and the right level of abstraction was sometimes hard to determine. This was partly due to the interconnectedness of content, i.e., descriptions of a series of events that all affected one another. For example, working in a context in which it feels possible to provide high-quality care to patients is a prerequisite for feeling both that one is working effectively and that one is supported by the organisation, and various organisational aspects might explain why nurses felt that they had no time for recovery.

Being aware of potential errors in the coding process, the authors checked and discussed coding precision during the analysis. As the units of analysis provided insight into experiences of work motivation, ITL, and departures from the nursing profession without direct interaction between the researchers and informants, interviewer bias could be avoided [42]. One obvious limitation was the lack of opportunity to ask follow-up questions to deepen contextual understanding. Another disadvantage was the absence of information from nurses who did not answer the open-ended questions. That said, postal surveys can be assumed to exert minimum pressure on respondents to give ‘socially acceptable' responses. So, it may have been easier for some groups to respond—for example, those with critical opinions, those who are more reticent or nurses on sick leave—and harder for others, such as nurses with reading and writing difficulties. The strength of the study was the large national sample with a wide range of experience gained in many parts of the healthcare sector, which improved the prospects of shedding light on a range of experiences relating to work motivation and turnover. The transferability of results is restricted by the fact that this is a “self-selected” subsample within the LANE study. However, by representing a subsample of the larger national LANE study, the context of the sample was well defined [21] and represents 46% of the nurses in the LANE study cohorts.

## 5. Conclusions

Although motivation, intention to leave the profession, and turnover are complex phenomena influenced by multiple factors, by exploring them together, it was possible to identify important clues as to how the profession might retain more experienced nurses. Based on the results of this study, we conclude that ensuring that nurses have a manageable workload, that they have autonomy, that their competence is utilised, and that they enjoy adequate support from management will go some way to addressing the forecast shortage of nurses, by promoting the wellbeing of and motivating experienced nurses and decreasing staff turnover. Furthermore, according to nurses with over ten years of professional practice, a sustainable working life is dependent on collegiality, being able to work in competent teams, and being afforded multiple career opportunities. When deciding whether to remain in or leave the profession, recognition of the demands of the profession and individual knowledge and skills in the form of a fair and reasonable salary are considered key factors. The result of this study also indicates that what may be considered hygiene factors in some contexts may function as motivation factors in nursing. Finally, the study makes clear that an organisation must be alert to and able to address the early signs of work-related ill health in its employees if it is to mitigate ITL and staff turnover.

## Figures and Tables

**Table 1:** The open-ended questions from the LANE survey and the number of answers in the form of written comments to each question (total number of comments 1,542).

Areas	Question in survey	Number of comments
Professional work motivation	*“If you have something else you would like to say about yourself or the study, you are welcome to write it here. Of particular interest are your views and experiences regarding what factors do you consider to be crucial for being engaged and motivated in the work in the long-term?”*	807

Intention to leave the profession	*“If you are thinking about leaving the profession—please state why below”* The open-ended question was preceded by three closed-ended questions on intention to leave the nursing profession including thoughts about leaving and leaving behaviour (i.e., 1. I think a lot about leaving the profession, 2. I am actively looking for another job outside the nursing profession, and 3. I will leave the nursing profession as soon as possible)	542

Exit: having left the profession	*“If you have chosen to leave the profession as a nurse/specialist nurse/midwife/other specialization, - if possible, please state why below:”*	193

**Table 2:** Example of the analysis process and verbatim quotes to each subcategory.

	Meaning unit	Code	Subcategory	Category
Motivation	*Having some say over daily working hours, duties and long-term shift arrangements is an important motivator*	Ability to control working hours	Influence over working hours	Organization characteristics
ITL	*The desire for a job that does not require that almost all off-duty time be used for recovery. A job without weekend or night shifts that allows me to spend weekends and holidays with my family and friends*	Call for work that enables private and family life	Working hours affect private and family life	Organization characteristics
Turnover	*I was no longer able to cope with constantly changing working hours and shifts, the sense that I never had time to get everything done, to do the job I was supposed to be doing*	Unsustainable scheduling	Difficulty to combine work with private life	Organization characteristics

Motivation	*That, as a nurse, you have the time and opportunity to develop knowledge and skills. A career ladder: which paths and opportunities are open to me? A good salary. Responsive managers that take matters seriously*	Opportunities for professional development in nursing	Development and challenges	Work characteristics
ITL	*Nursing is a demanding and responsible career that can be burdensome at times. Even at home, it can be difficult to stop thinking about work*	Demanding and distressing work	Emotional and physically distressing work	Work characteristics
Turnover	*There is no time to be the nurse I want to be. Sometimes I am completely drained after having responsibility for almost 50 patients*	No chances to perform the care I believe in	No opportunity for nursing care	Work characteristics

Motivation	*The most important factor for engendering commitment and motivation is a pleasant work climate: understanding and helpful colleagues, and a manager who is responsive, fair, sympathetic, humble and capable of leading the team*	Pleasant work climate	Well-functioning team collaboration	Relationships at work
ITL	*Does not enjoy the job because of the high workload and poor climate between colleagues, and between colleagues and management*	Bad collegial climate	Lack of teamwork	Relationships at work
Turnover	*I had no possibilities to influence my work situation and the work environment was too hierarchical*	Hierarchies in healthcare	Hierarchical collegial structure	Relationships at work

Motivation	*I consider appreciation of the work one does to be vital to wellbeing. The most important factor is an adequate salary. This is why I have worked as a travel nurse for the last two and a half years. This provides me with a good salary. I can influence my own working hours and I am appreciated at work every day*	Good and fair salary	Fair salary	Recognition
ITL	*In my current workplace, there is little possibility to affect my salary even if I continuously develop my competence and take on greater responsibility*	Little opportunity to affect salary	No influence on the salary	Recognition
Turnover	*The working hours and high workload cause ill health. I no longer wish to work for the regional health authority because of the poor pay and dreadful salary development*	Poor pay and salary development	Not the right salary for the job performed	Recognition

Motivation	*—*	*—*	*—*	*—*
ITL	*The stress of my job affects me physically. I don't want to become ill with stress. I would like to have a job without responsibility for other people's health. The responsibility is too great*	Worries about the own health	Fear of developing health issues	Health issues
Turnover	*Stress and the dreadful working conditions, with such weak leadership, made me ill […]. I didn't see how I could ever be well as long as I remained in the profession*	Didn't think I could stay and get well	Health negatively affected	Health issues

## Data Availability

Data obtained for the study will not be accessible to others due to survey respondents being assured raw data would remain confidential.
